# Cell density-dependent differential proliferation of neural stem cells on omnidirectional nanopore-arrayed surface

**DOI:** 10.1038/s41598-017-13372-6

**Published:** 2017-10-12

**Authors:** Kyoung Je Cha, Sun-Young Kong, Ji Soo Lee, Hyung Woo Kim, Jae-Yeon Shin, Moonwoo La, Byung Woo Han, Dong Sung Kim, Hyun-Jung Kim

**Affiliations:** 1 Department of Mechanical Engineering, Pohang University of Science and Technology (POSTECH), San 31 Hyoja-dong Nam-gu, Pohang, 790-784 South Korea; 2 Laboratory of Molecular and Stem Cell Pharmacology, College of Pharmacy, Chung-Ang University, 221 Heukseok-dong Dongjak-gu, Seoul, 156-756 South Korea; 3 Department of Biochemistry, College of pharmacy, Seoul National University, San 56-1 Sillim-dong Gwanak-gu, Seoul, 151-742 South Korea; 4 Ultimate Fabrication Technology Group, Korea Institute of Industrial Technology (KITECH), Techno sunhwan-ro Yuga-myeon Dalseong-gun, Deagu, 711-880 South Korea; 5 Molds & Dies R&D Group, Korea Institute of Industrial Technology (KITECH), 156 Gaetbeol-ro, Yeonsu-gu, Incheon, 406-840 South Korea

## Abstract

Recently, the importance of surface nanotopography in the determination of stem cell fate and behavior has been revealed. In the current study, we generated polystyrene cell-culture dishes with an omnidirectional nanopore arrayed surface (ONAS) (diameter: 200 nm, depth: 500 nm, center-to-center distance: 500 nm) and investigated the effects of nanotopography on rat neural stem cells (NSCs). NSCs cultured on ONAS proliferated better than those on the flat surface when cell density was low and showed less spontaneous differentiation during proliferation in the presence of mitogens. Interestingly, NSCs cultured on ONAS at clonal density demonstrated a propensity to generate neurospheres, whereas those on the flat surface migrated out, proliferated as individuals, and spread out to attach to the surface. However, the differential patterns of proliferation were cell density-dependent since the distinct phenomena were lost when cell density was increased. ONAS modulated cytoskeletal reorganization and inhibited formation of focal adhesion, which is generally observed in NSCs grown on flat surfaces. ONAS appeared to reinforce NSC-NSC interaction, restricted individual cell migration and prohibited NSC attachment to the nanopore surface. These data demonstrate that ONAS maintains NSCs as undifferentiated while retaining multipotency and is a better topography for culturing low density NSCs.

## Introduction

Neural stem cells (NSCs) have the capacity to self-renew and differentiate into neurons, astrocytes, and oligodendrocytes, and play an important role as promising cells to treat neurodegenerative diseases and central nervous system injuries^1–3^. Precise control of NSC proliferation without losing multipotency and differentiation in order to generate specific cell types is a key issue in stem cell biology and regenerative medicine. Chemical cues with soluble diffusible molecules or molecules bound to extracellular surfaces are already well accepted by many biologists to regulate differentiation and proliferation *in vitro* ^4–6^. For example, extrinsic factors such as epidermal growth factor (EGF), fibroblast growth factor 2 (FGF2), retinoic acid and ciliary neurotropic factor are known to regulate cell proliferation or differentiation^7–11^. Intrinsic factors that play important roles in the generation of neurons, astrocytes and oligodendrocytes have been studied^12,13^, but the culture conditions and methods to generate functional cell types have not yet been fully identified. In addition, the guided differentiation of stem cells by chemical or biological factors often requires a relatively long time (weeks to months) and high cost for cell culture. These have limited the widespread use of stem cell therapy and applications. Hence, there is a definite need to develop efficient methods for controlling the proliferation and differentiation of NSCs.

During the process of cell fate determination, stem cells undergo a number of morphological alterations by physical cues from cell surface substrates as well as chemical factors in the culture media^14^. Intriguingly, stimuli that trigger changes in cell morphologies have been shown to regulate stem cell differentiation^15–17^. Physical cues acting on stem cells *in vivo* include the micro/nano-topographic features and mechanical properties of the extracellular matrix (ECM), whose effects cannot be expected on the flat surfaces in general culture systems. It is now well accepted that nanotopography mimicking nanostructures of well-defined ECM assists to promote tissue-specific cell function *in vitro*. The reason for introducing nanotopography is to facilitate restoration of physiological functions and structures, such as migration, alignment, differentiation and proliferation, by providing naturally occurring microenvironments. Thus, surface topography has recently received great attention as a biomaterial to be used for regulating stem cell behaviors^18–20^.

Previous studies showed that nanotopography influences stem cell biology considerably by changing protein adsorption, filopodia production, focal adhesion formation, nucleus shape, and cytoskeletal contraction^21–23^. In addition, there are some promising results with respect to the promotion of neurogenesis^24,25^. However, most reports regarding the effects of nanotopography during NSC culture are from unidirectionally aligned nanograting or nanofiber structures^26–31^. Previous studies have shown that the aligned nanostructures are effective not only in promoting neurogenesis but also in enhancing neurite (axon and dendrites) outgrowth guidance *in vitro*. It may be the radial processes of radial glial cells that are essential for the guidance of neuronal migration and alignment, and neurite outgrowth^32^.

To elucidate the underlying mechanism of cell response to mechanical stimuli, such as nanotopography, reproducible nanofabrication techniques are needed, in particular the use of polystyrene (PS), which has been widely used as a substrate material of cell-culture wares over several decades. Recently, we have developed a PS cell-culture platform where self-ordered hexagonal arrays of nanopores are patterned on the surface, referred to as a nano Petri dish, for a cell-nanotopography interaction study^33^. The nanopore-arrayed surface was found to regulate various cell functions such as cell attachment, proliferation and differentiation^33–35^.

NSCs are cultured as either free-floating aggregates, known as neurospheres, or as a monolayer^9,36^. Culture dish coating materials, including polyornithine, laminin, poly-D-lysine (PDL), poly-L-lysine, and fibronectin, enable NSCs to grow as monolayer on the PS culture plates^9,37^. However, without coating with such substrates, NSCs generally do not attach to the PS surface, thus NSCs float in the media and proliferate as neurospheres when growth factors such as FGF2 and/or EGF are supplemented. Until recently, NSC culture conditions were mainly obtained from flat surfaces. Thus, in the present study, to explore the effects of nanotopography on proliferation, differentiation, and migration properties of NSCs, we generated PS cell-culture dishes with an omnidirectional nanopore arrayed surface (ONAS) (diameter: ~200 nm, depth: ~500 nm, and center-to-center distance: ~500 nm) and investigated the effects of nanotopography on NSCs. We found that ONAS facilitated NSC proliferation when cell density was low. At low density, the pattern of NSC proliferation observed on ONAS was different from that on the flat surface. NSCs cultured on ONAS aggregated to form, and migrated as, neurospheres, whereas NSCs cultured on the flat surface proliferated and migrated individually. These data suggest a regulatory role of topography in NSC proliferation and migration.

## Results

### Production of cell culture dishes with ONAS

**Figure 1:**
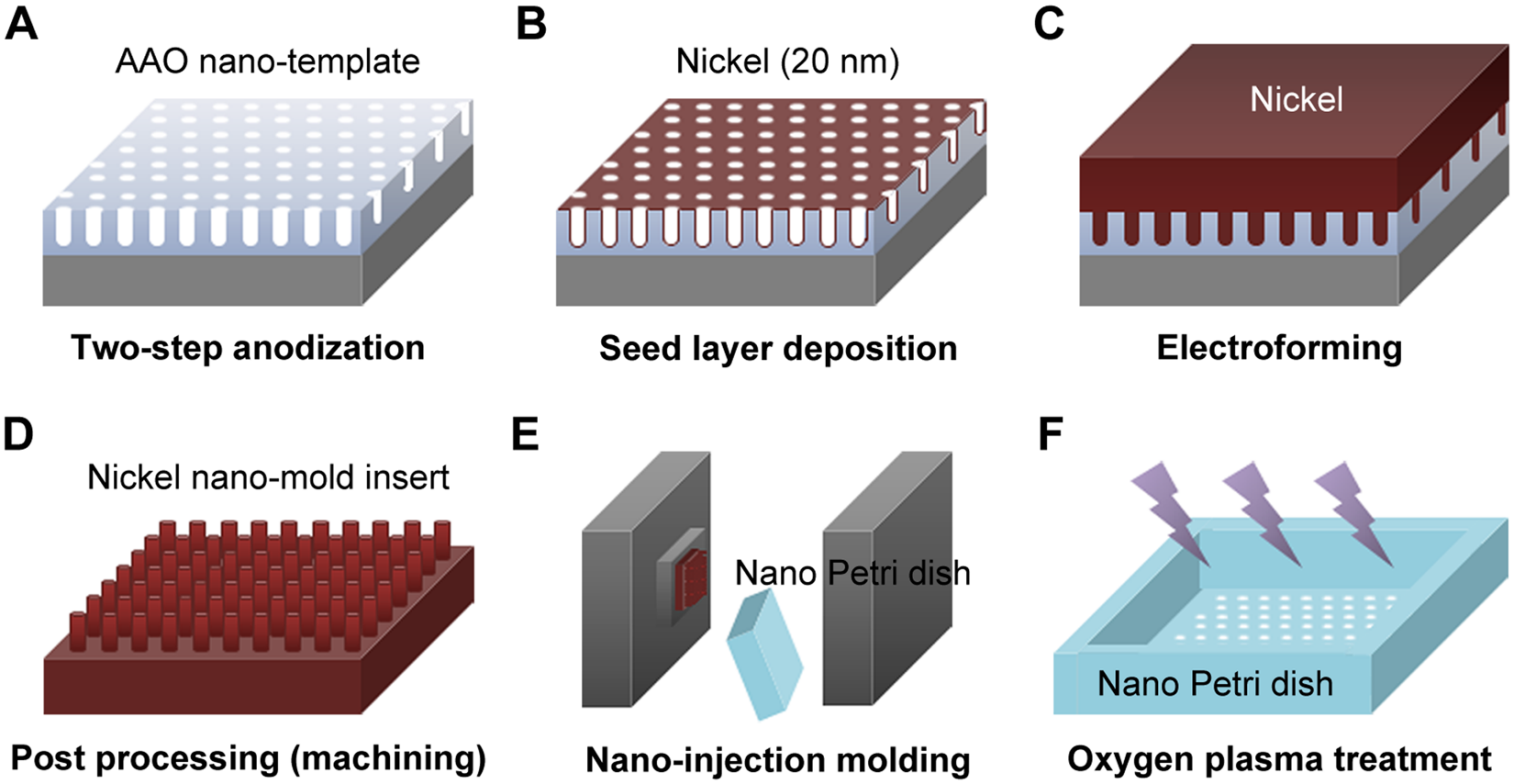
Schematic diagram of the fabrication process of PS cell-culture dishes with ONAS. (**A**) Two-step anodization of an aluminum substrate to fabricate an AAO nano-template. (**B**) Nickel seed layer deposition by electron beam evaporation to make a conductive base form (mandrel). (**C**) Nickel electroforming to manufacture a nickel nano-mold insert. (**D**) Post processing of the nano-mold insert to assemble into a mold base. (**E**) Nano-injection molding to replicate PS nano Petri dishes with ONAS. (**F**) Oxygen plasma treatment of the nano Petri dish to enhance hydrophilicity and cell adhesion.

The PS cell-culture platform with ONAS was fabricated as recently reported through a three-step process of two-step aluminum anodization, nickel electroforming and nano-injection molding, as shown in Fig. 1 ^20,33,34^. Briefly, an anodic aluminum oxide (AAO) nano-template with ONAS, which functions as a master template during the nickel electroforming process, was fabricated through a two-step aluminum anodization process (Fig. 1A). An ONAS on the AAO nano-template was formed into the desired dimension in a phosphoric acid bath by controlling several processing parameters described in the method section. A nickel nano-mold insert, which contains nanopillar arrays that are complementary in shape to the ONAS, was manufactured by an electroforming process on the AAO nano-template (Fig. 1B–D). A 20 nm-thick nickel seed layer was deposited on the nano-template surface by means of an electron beam evaporator to have uniform conductivity. The electroforming process was carefully performed in a nickel sulfamate bath by controlling electric current densities up to the desired thickness to minimize internal stress of the electrodeposited nickel layer. The electroformed part was then machined and polished to make a nano-mold insert with the nanopillar arrays over an area of 2 × 2 cm^2^. The nano Petri dishes with the ONAS were produced by a nano-injection molding process with the nano-mold insert (Fig. 1E). The area of ONAS in the nano Petri dishes would be further increased by improvement and optimization of fabrication process for nickel nano-mold insert, which is composed of AAO process followed by nickel electroforming, and nano-injection molding process for replication of nano Petri dishes.

**Figure 2:**
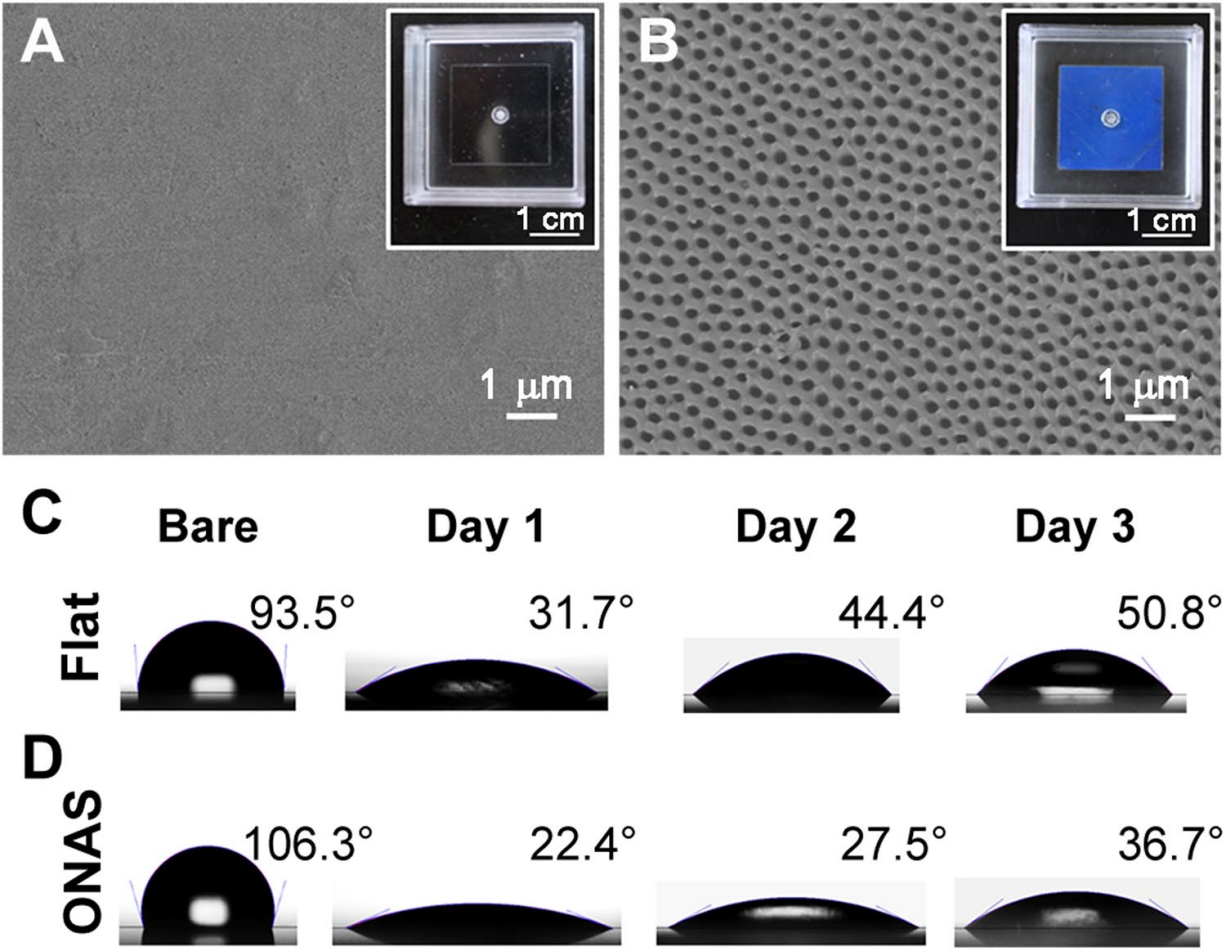
Representative images of flat surface and ONAS and the alteration of contact angles by oxygen plasma treatment. Scanning electron microscope images and photographs of the nano-injection molded flat surface (**A**) and ONAS (**B**) on PS flat and nano Petri dishes, respectively. Contact angle measurements of sessile water droplets on the flat surface (**C**) and ONAS (**D**), with and without oxygen plasma treatment.

Conventional PS flat-surface Petri dishes, used as a control group, were also produced by injection molding with a mold insert having a mirror-like surface. Figure 2A and B show scanning electron microscope (SEM) images of the replicated PS flat and nanopore-arrayed surfaces. After oxygen plasma treatment, water contact angles (CAs) of flat surface and ONAS were measured (Fig. 2C and D). The untreated hydrophobic surfaces were found to have CAs over 90°, whereas the oxygen plasma-treated surfaces showed CAs less than 60°.

### ONAS increases proliferation and decreases spontaneous differentiation of NSCs

**Figure 3:**
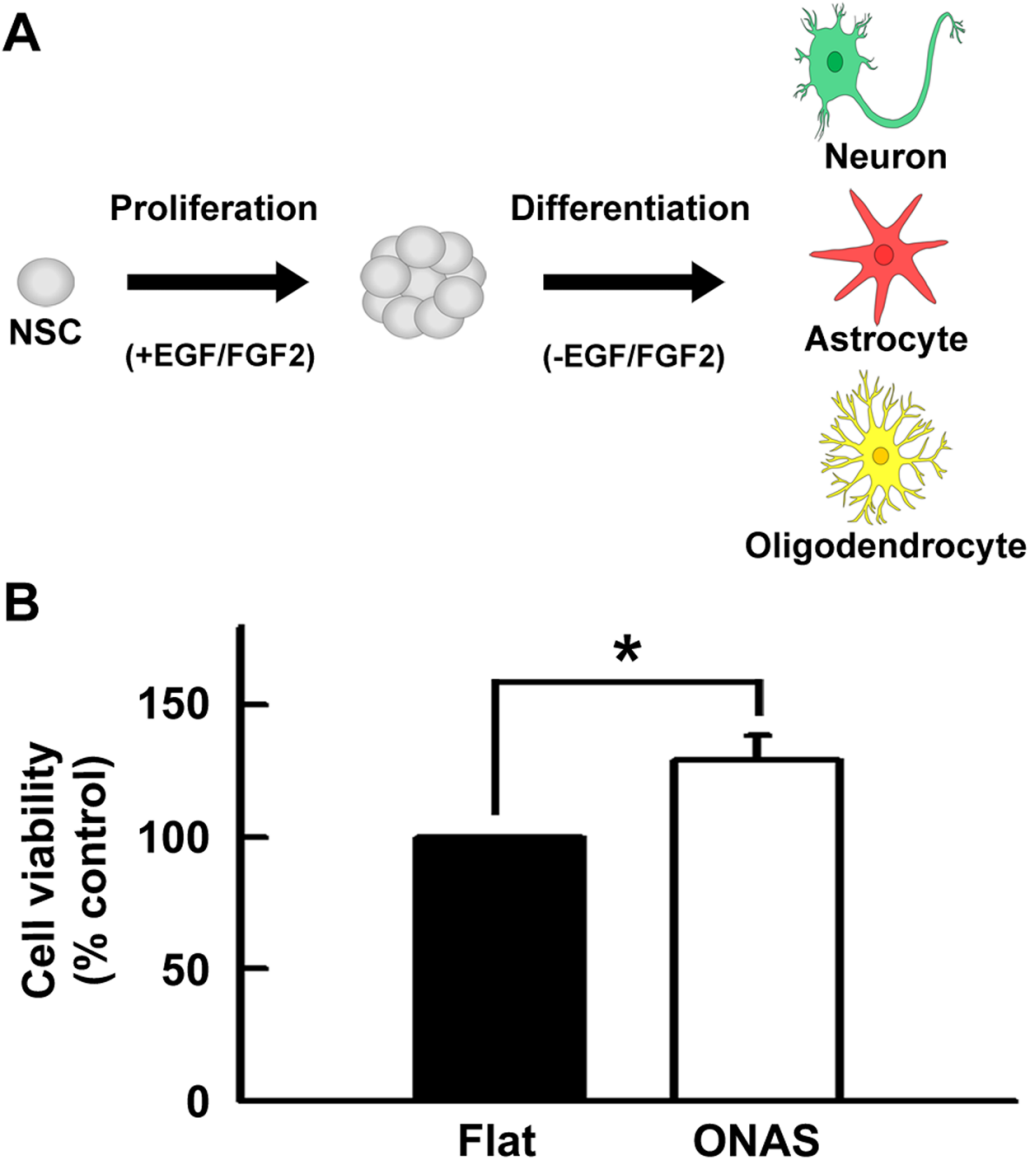
ONAS increases the numbers of NSCs in the presence of EGF and FGF2. (**A**) NSCs proliferate in the presence of EGF and FGF2 and differentiate in the absence of mitogens. NSCs can differentiate into neurons, astrocytes and oligodendrocytes. (**B**) Determination of cell viability. After 1 week of expansion in the presence of EGF and FGF2, NSCs were dissociated and plated onto either the flat surface or ONAS. NSCs were induced to proliferate for an additional day in the presence of mitogens and cell viability was assessed by MTT assay. Values were mean ± s.e.m. (n = 3). Statistical analysis of data was performed using the Student’s *t*-test (*P < 0.05).

To explore the influence of ONAS on NSC culture, we plated NSCs on either flat surface or ONAS. NSCs proliferate in the presence of EGF and FGF2 and differentiate when growth factors are withdrawn from the media (Fig. 3A)^38^. Neural cells are not easy to culture; one of the reasons is that these cells do not attach to the surface of PS plates. Generally PDL, laminin, polyornithine and/or fibronectins are used to increase neural cell attachment. Dissociated NSCs that are derived from the cortex of E14 rats are cultured on PDL- and laminin-coated culture plates to increase adherence to the bottom of plates. To test whether ONAS enhances NSC attachment to the surface of plates, we cultured NSCs on either a flat surface or ONAS at a density of 2 × 10^4^ cells cm^−2^ in the presence of EGF and FGF2 without PDL or laminin coating. When NSCs were observed under a microscope to identify attached cells, we found that ONAS was not sufficient to increase NSC attachment (data not shown). Most cells detached from the bottom and floated in the media suggesting that ONAS does not facilitate NSC attachment.

**Figure 4:**
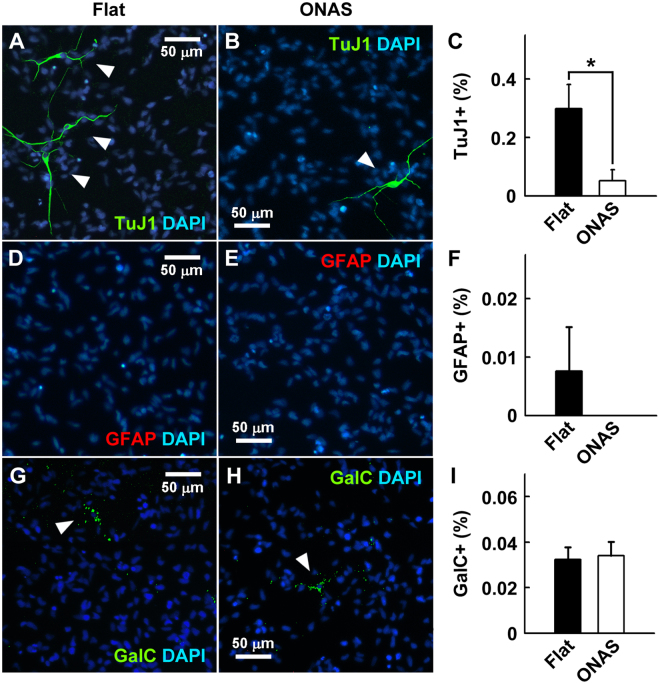
ONAS reduces spontaneous differentiation of neurons during NSC proliferation. (**A–I**) NSCs were expanded for 1 week as neurospheres and dissociated onto the flat surface or ONAS for 3 additional days in the presence of EGF and FGF2. Then, NSCs were fixed and immunostained with TuJ1 antibody (**A–C**, green), anti-GFAP antibody (**D–F**, red) or anti-GalC antibody (**G–I**, green) to detect neurons, astrocytes or oligodenrocytes, respectively. Nuclei were stained with DAPI (blue). Scale bar, 50 μm. TuJ1-positive (**C**), GFAP-positive (**F**) or GalC-positive cells (**I**) were counted and normalized to total cell number to obtain percentage. Data were presented as mean ± s.e.m. (n = 3). Statistical analysis of data was performed using the Student’s *t*-test (*P < 0.05).

Next, to investigate whether ONAS affects proliferation of NSCs, we cultured NSCs on PDL and laminin coated flat surface  or ONAS at a density of 2 × 10^4^ cells cm^−2^ in the presence of EGF and FGF2. One day after plating, the levels of surviving cells were assessed by MTT assay. As shown in Fig. 3B, ONAS significantly enhanced the NSC proliferation by 29.16% (P < 0.05) compared to the flat surface. However, the MTT values of ONAS on the 3^rd^ and 4^th^ days were not different from those of the flat surface suggesting that ONAS induces proliferation of NSCs in the early days or only when the cell density is low (data not shown). When NSCs were immunostained after 3 days of expansion in the presence of EGF and FGF2 to identify cells that had spontaneously differentiated, we found significantly less TuJ1 (antibody for pan neuronal marker protein βIII Tubulin) positive neurons (P < 0.05) in the NSCs grown on ONAS when compared with those on the flat surface (Fig. 4A–C). The amounts of spontaneous differentiation of astrocytes or oligodendrocytes were not different in NSCs cultured on either flat or ONAS (Fig. 4D–I). Altogether, these data suggest that the initial proliferation of NSCs is enhanced and spontaneous neuronal differentiation is reduced in the presence of mitogens when NSCs were cultured on ONAS.

### ONAS does not change the multipotency of NSCs during differentiation

**Figure 5:**
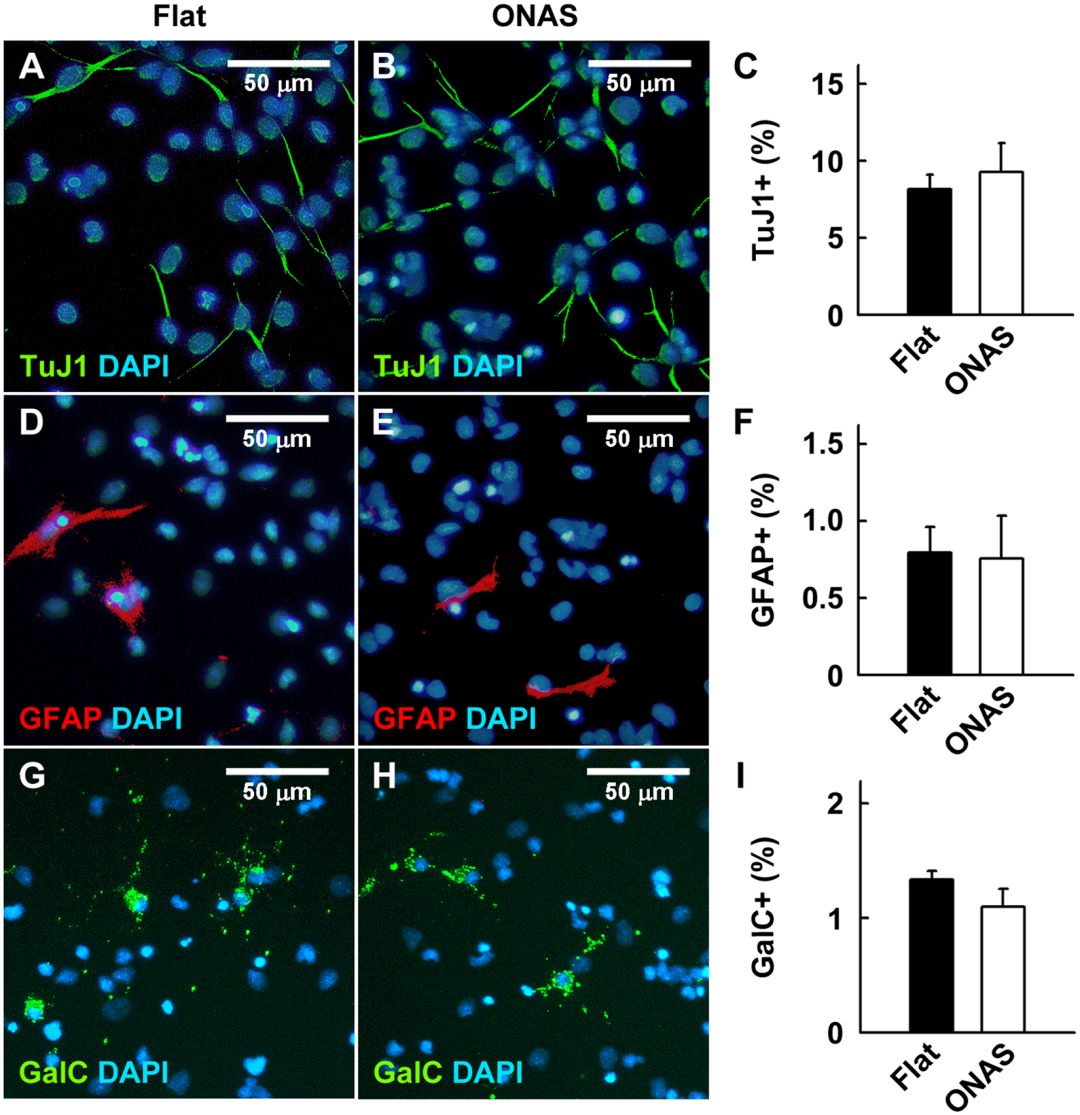
ONAS does not change multipotency of NSCs during differentiation. (**A–I**) NSCs were expanded for 1 week as neurospheres, dissociated onto the flat surface or ONAS, and differentiated for 4 days in the absence of EGF and FGF2. Then, cells were fixed and immunostained with TuJ1 antibody (**A–C**, green), anti-GFAP antibody (**D–F**, red) or anti-GalC antibody (**G–I**, green). Nuclei were stained with DAPI (blue). Scale bar, 50 μm. Quantification of neurons (**C**), astrocytes (**F**) and oligodendrocytes (**I**). TuJ1-positive, GFAP-positive or GalC-positive cells were counted and normalized to total cell number to obtain percentage. The values were presented as mean ± s.e.m. (n = 4 for neurons and astrocytes, n = 3 for oligodendrocytes). Statistical analysis of data was performed using the Student’s *t*-test.

NSCs differentiate into neurons and glia when growth factors are withdrawn from the media (Fig. 3A). To explore the role of ONAS in NSC fate control during differentiation, we plated NSCs onto either the flat surface or ONAS and differentiated them in the absence of EGF and FGF2 for 4 days. After fixation, cells were immunostained for neurons, astrocytes and oligodendrocytes using TuJ1, anti-GFAP antibodies and anti-GalC antibody, respectively. As shown in Fig. 5, no significant differences (P > 0.05) in the percentage of TuJ1+, GFAP+ and GalC+ cells were observed between NSCs cultured on the flat surface and those on ONAS. These data suggest that ONAS does not induce the differentiation of specific cell types and does not alter NSC multipotency during differentiation.

### NSCs grow as spheres on ONAS when plated at clonal density

**Figure 6:**
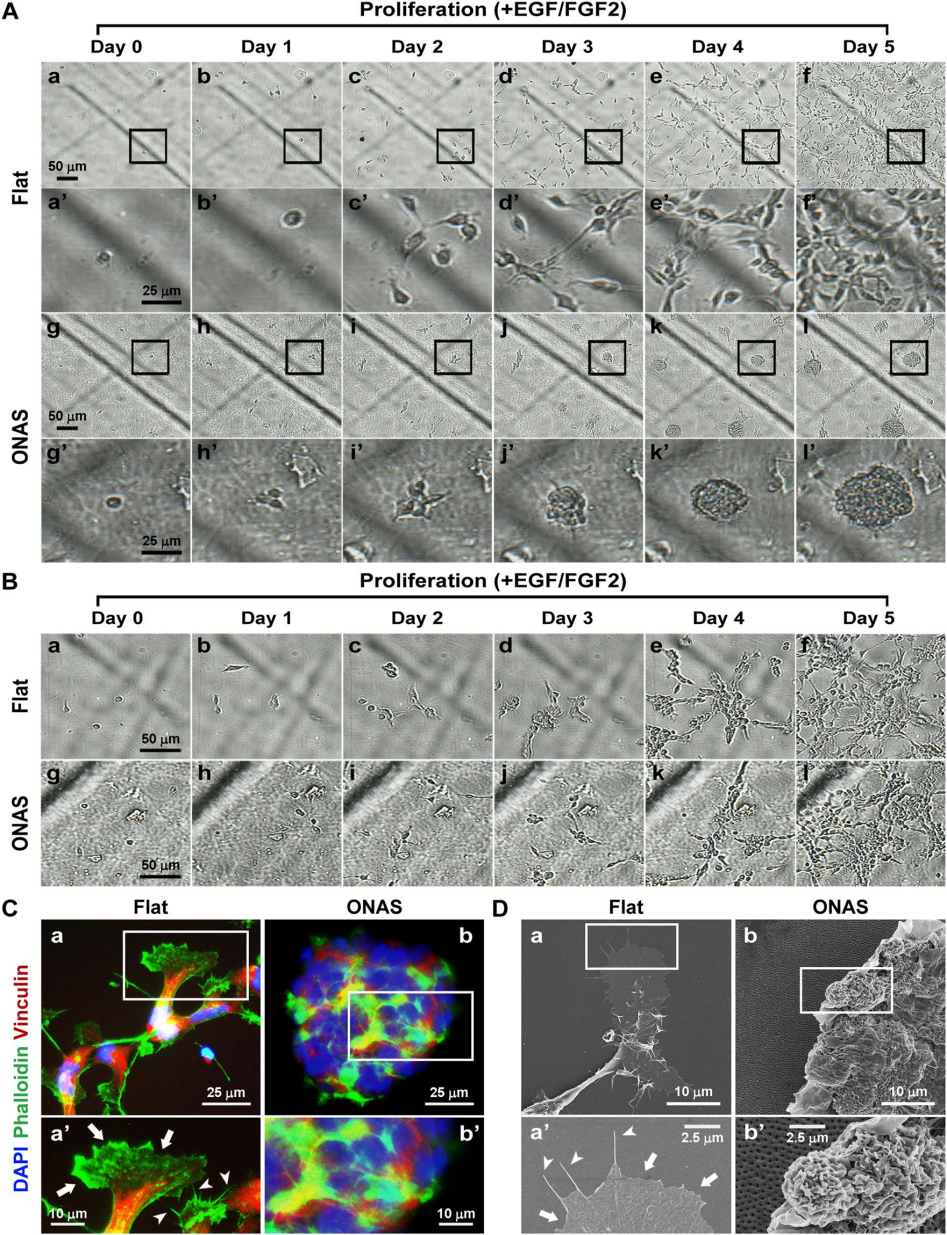
NSCs on ONAS show a distinct proliferation pattern when cultured at clonal density (3.3 cells μl^−1^). (**A**) Time-lapse digital images of NSCs grown in the presence of mitogens on either the flat surface (a–f, a’–f’) or ONAS (g–l, g’–l’) at 3.3 cells μl^−1^. Boxed area in a-l is enlarged in a’–l’. The each scale bar is 50 μm in a–l and 25 μm in a’–l’. (**B**) Time-lapse digital images of NSCs cultured in the presence of EGF and FGF2 on flat surface or ONAS at 6.7 cells μl^−1^. Scale bar, 50 μm. (**C**) NSCs grown in the presence of mitogens for 4 days were fixed and stained with phalloidin (green) and anti-vinculin antibody (red). Nuclei were stained with DAPI (blue). Boxed areas in a and b were enlarged in a’ and b’. Arrows and arrowheads in a’ indicate lamellipodia and filopodia, respectively. Scale bar, 25 μm (in a and b) and 10 μm (in a’ and b’). (**D**) Field emission scanning microscopy photos of NSCs cultured on Flat or ONAS. NSCs grown in the presence of mitogens for 4 days were fixed and photos were taken. Boxed areas (a and b) were enlarged in a’ and b’. Arrows and arrowheads in a’ indicate lamellipodia and filopodia, respectively. Scale bar, 10 μm (in a and b) and 2.5 μm (in a’ and b’).

Since we found induction of NSC proliferation only on the first day (Fig. 3B), we hypothesized that ONAS may facilitate proliferation when cell density is low. Thus, we dissociated and plated NSCs at 3.3 cells μl^−1^ (1111 cells cm^−2^) which is considered as clonal density on either flat or nanopore surfaces. Interestingly, we observed differential proliferation patterns of NSCs depending on the surface conditions. NSCs seeded on flat surfaces increased cell numbers and spread on the surface (Fig. 6A). In contrast, those on ONAS aggregated to form neurospheres (Fig. 6A). In order to confirm the proliferation pattern of NSCs on ONAS, we recorded time-lapse video (Supplementary Videos S1 and S2). NSCs were dissociated and seeded on either flat or ONAS plates for 24 h to allow cells to attach and stabilize, and the images were acquired every 10 min for an additional 48 h. When plated at clonal density (3.3 cells μl^−1^, 1111 cells cm^−2^), NSCs grown on the flat surface had a tendency to find each other and divide, and each cell migrated out independently (Supplementary Video S1 and Supplementary Fig. S1A). In contrast, NSCs seeded on ONAS aggregated to form neurospheres and proliferated, and they stuck to each other and moved around as a neurosphere (Supplementary Video S2 and Supplementary Fig. S1A). Interestingly, the phenomena were density-dependent. When we plated cells at twice more of the density (6.7 cells μl^−1^, 2222 cells cm^−2^), the phenomena of induction of the neurosphere formation disappeared (Fig. 6B). Immunostaining with phalloidin and anti-vinculin antibody enabled visualization of F-actin and focal adhesions, respectively. NSCs cultured on the flat surface showed lamellipodia, filopodia and stress fibers, however, those on ONAS rounded up as neurospheres and the cytoskeleton of individual cells was difficult to recognize (Fig. 6C and Supplementary Fig. S1B and C). With field emission SEM (FE-SEM), we also observed formation of lamellipodia and filopodia in NSC culture on flat surface (Fig. 6D). However, NSCs on ONAS attached to other neighboring NSCs when photos were taken by FE-SEM (Fig. 6D). These data suggest that ONAS influences NSC proliferation only when cells are plated at low density.

**Figure 7:**
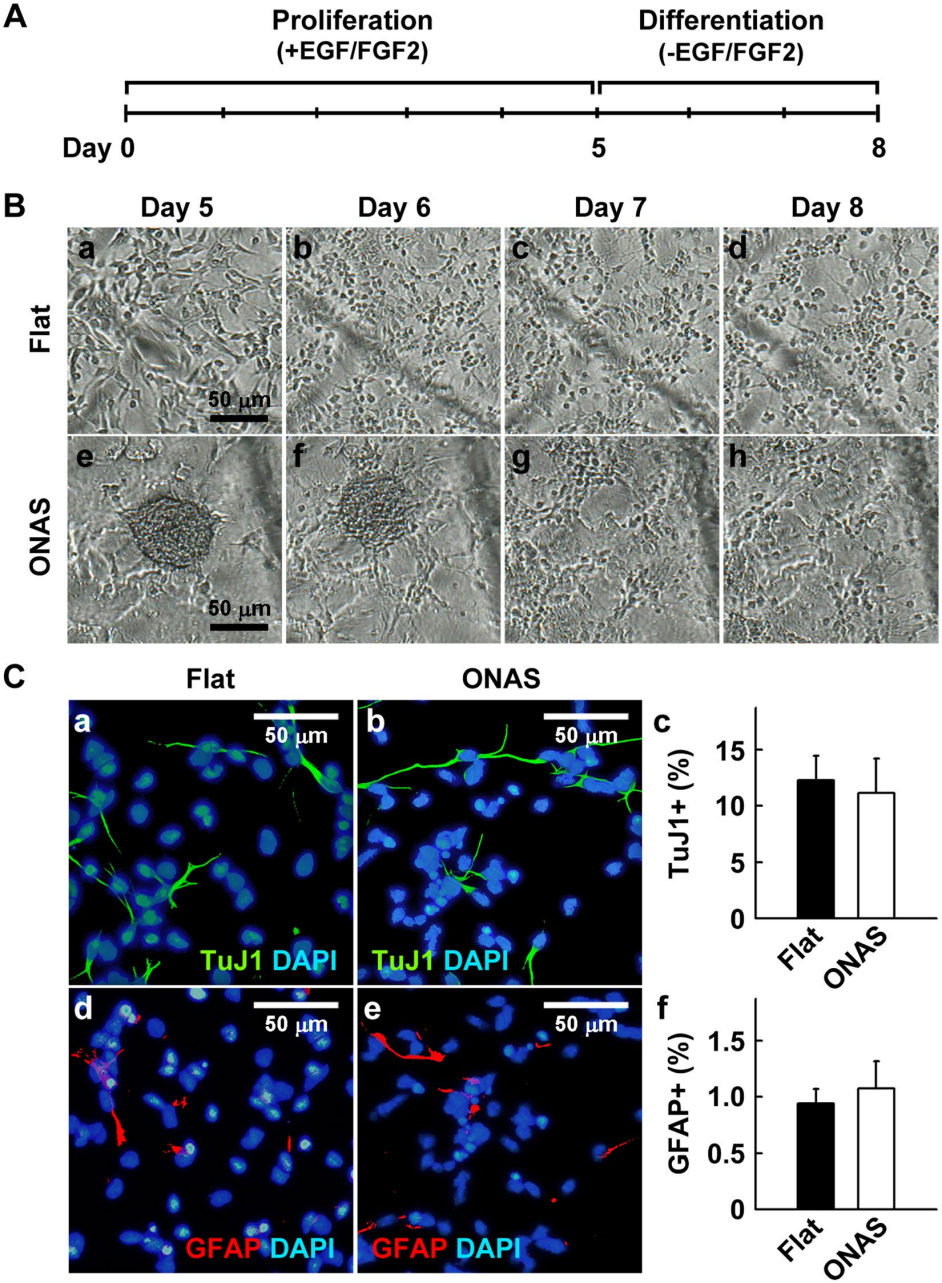
ONAS does not change multipotency of NSCs (3.3 cells μl^−1^) during differentiation. (**A**) Experimental scheme. NSCs (3.3 cells μl^−1^) were cultured in the presence of mitogen for 5 days and differentiated for 3 additional days in the absence of EGF and FGF2. (**B**) At the 5^th^ day of proliferation at 3.3 cells μl^−1^, NSCs on the flat surface grew individually, whereas NSCs on ONAS formed neurospheres. After 3 additional days of differentiation, NSCs on ONAS migrated out from the neurosphere and differentiated. Scale bar, 50 μm. (**C**) NSCs (3.3 cells μl^−1^) that proliferated differentially on the flat surface or ONAS were differentiated for 3 additional days, fixed and immunostained with TuJ1 antibody (a and b, green) and anti-GFAP antibody (d and e, red). Scale bar, 50 μm. Quantification of neurons (c) and astrocytes (f). Data were expressed as mean ± s.e.m. (n = 3). Statistical analysis was performed using the Student’s *t*-test.

Since NSCs at clonal density showed a unique proliferation pattern, we decided to explore whether NSCs grown as neurospheres on ONAS possess altered multipotency. After five days of proliferation on either the flat surface or ONAS in the presence of mitogens, NSCs were allowed to differentiate for 3 additional days in the absence of EGF and FGF2 (Fig. 7A and B). After fixation, cells were immunostained for neurons and astrocytes using TuJ1 and anti-GFAP antibodies, respectively. As shown in Fig. 7C, no significant difference in the percentage of TuJ1+ (P = 0.3885) and GFAP+ (P = 0.3275) cells was obtained between NSCs differentiated on the flat surface and those on ONAS. These data suggest that the proliferating pattern of NSCs was changed, but the capacity of NSCs to differentiate into neurons and astrocytes was not altered by ONAS when NSCs were cultured at low density.

## Discussion

In the current study, the nano Petri dishes with ONAS were prepared to investigate the nanotopographical effects on migration, proliferation, and differentiation of NSCs. The nano injection molding process was applied to replicate a large number of the same dishes in a short time (the overall fabrication time of a single nano Petri dish was ~30 s). As described earlier, the mass-producible nano Petri dish has several advantages for cell-nanotopography interaction studies since many of the same samples are required at the transition stage from basic research to clinical applications. PS, used as the substrate material, has stable surface properties after oxidation and high biocompatibility and has been proven for cell-culture wares and biological applications during past several decades.

Although the detailed mechanisms involved in stem cells’ response to nanotopography are not identified, others also reported the effects of nanostructure on NSCs or other types of stem cells. Human ES cells (hESCs) differentiated into neurons when plated on a 350 nm ridge/groove pattern for 5 days, even without the addition of exogenous soluble factors^29^. A hexagonal or honeycomb nanopillar structure up-regulated and maintained Oct4 expression in hESCs in the absence of FGF2^39^. Mouse ESCs not only differentiated into the neuronal lineage but also enhanced neurite outgrowth on electrospun aligned, uniaxial nanofibers^28^. When Yang *et al*. used diverse nanoscale dimensions (ranging from 300 to 1500 nm groove width and pillar diameter/gap) to investigate the effects of topographical cues on hNSC differentiation, they found that the smaller nanostructures (300–300 nm groove ridges and 300–300 nm pillar diameter/gaps) effectively enhanced formation of focal adhesion complexes and induced hNSCs to differentiate toward neurons and astrocytes^31^. In addition, it is reported that highly aligned, laminin-coated PS nanofiber mesh promoted neurogenesis in rat NSCs^30^. Depending on the size, morphology, height, and depth of the nanostructure, the differentiation, migration, fate and proliferation of stem cells appeared to be affected.

The control of cell microenvironments *via* nanotopographical cues may improve not only to elucidate the influence of topographical stimuli on stem cell fate/functions but also to design cell culture-wares for the generation of specific types of differentiated cells for cell therapy. Extrinsic factors that are known to modulate stem cell fate and proliferation are expensive. Thus, it would be more economical to modulate nanotopography by production of culture-wares that bear nanosurfaces than to use cytokines or growth factors to regulate stem cell fate or behavior.

When NSCs were cultured on ONAS made of PS, we did not observe any changes in NSC fate determination but found that the nanopore structure inhibited spontaneous differentiation while increasing early NSC proliferation. It appears that the effects of nanostructure are distinctive depending on the types of cells since our recent report showed that ONAS promoted pancreatic differentiation by increasing the expression of pancreatic progenitor marker PDX1 in hESCs and induced pluripotent stem cells^20^. However, in the current study, NSCs plated on ONAS appeared to maintain the key stem cell characteristics such as self-renewal and possession of differentiation potential better than those cultured on flat surfaces without controlling cell fate. Since NSCs and other stem cells respond to external stimuli, and the properties and fate of each stem cell can constantly be changed by extrinsic factors or autocrine/paracrine factors^2,3,40–49^, it is hard to obtain data from homogenous populations of stem cells or NSCs. However, culturing NSCs on ONAS appears to provide homogenous NSC samples since NSCs show fewer tendencies of spontaneous differentiation on the nanosurface. Therefore, more accurate transcriptome data of homogeneous NSCs can be obtained by culturing NSCs on ONAS.

Just 1 day after plating, we observed an increase of NSC proliferation on ONAS. However, interestingly, the induction of proliferation disappeared from the 2^nd^ day after plating, suggesting that ONAS induces NSC proliferation only when cell density is relatively low. Since cells are closely located on ONAS when plated at low density, the paracrine factors released from cells may affect neighboring – attached NSCs on ONAS more than cells located in a distance on flat surface and may facilitate NSC proliferation on ONAS. NSCs are not easy to culture at low density or as single cells, and it has been reported that at least certain numbers of NSCs should be present to facilitate proliferation^50^. However, when NSCs are cultured at high density as neurospheres, it is possible to get hybrid or merged neurospheres that result in a heterogeneous population of differentiated cells^51^. Since it is hard to control precise differentiation due to the heterogeneity of cells in high-density culture, lots of effort has been made to culture NSCs at low density. For clonal culture, cells at 10 cells μl^−1^ or less are plated for neurosphere culture^51–53^. However, van der Kooy and his colleagues reported that even looking at neurosphere cultures at clonal density during experiments can result in chimera-sphere-formation, which may not be truly clonal^51^. Since NSC proliferation increased when the cell density is low and spontaneous differentiation was prevented on ONAS, it is advantageous to culture NSCs on ONAS to obtain more numbers of less differentiated NSCs. In addition, ONAS may be useful to culture amount limiting NSCs such as patient derived NSCs or disease model derived NSCs for the early phase culture to expand the numbers of them.

We obtained intriguing data when we cultured NSCs as neurospheres for 1 week then dissociated cells and plated on ONAS at 3.3 cells μl^−1^, which is known as clonal density, in the presence of EGF and FGF2. Although NSCs were plated as single cells, NSCs proliferated as neurospheres on ONAS. The NSCs divided but stuck together and did not migrate out on the nanopore surface. In contrast, NSCs cultured on the flat surface divided and migrated out to explore the surface and spread out. This phenomenon disappeared when NSCs were plated twice more (6.7 cells μl^−1^). Both NSCs seeded on ONAS and the flat surface divided and migrated around as individuals when plated at a density of 6.7 cells μl^−1^, suggesting that the effect of the ONAS is dependent on cell density. Like our results, it has been reported that hESCs cultured on nanopillar (diameter 120–170 nm) showed epithelial features and did not migrate much and rather formed colonies with neighboring hESCs and maintained their undifferentiated status^54^. Similarly, ONAS had tendency to hold cells together and allowed NSCs to migrate less than those plated on flat surface when cells were plated at low density, indicating that ONAS provides unfavorable environments for cell migration. Although it is unclear what factors are controlling the differential proliferation patterns, it is suggested that rat NSCs release cytokines, growth factors or micro-environmental vesicles that may act in autocrine or paracrine ways^47,55^. Some of researchers have noticed the role of autocrine/paracrine factors in NSC proliferation and/or differentiation^8,47^. Similar to our results, in their studies, it was observed that NSCs proliferated in a cell density-dependent manner even in the absence of FGF2 suggesting that paracrine/autocrine factors are involved in the NSC expansion. Others have reported that platelet-derived growth factor (PDGF) BB is one of the factors detected both in the lysates of neural progenitor cells (NPCs) derived from the cortex of rat E15 and in their conditioned media and delayed maturation of neurons during differentiation of NPCs^47^. In addition to PDGF, it has been known that multiple cytokines including interleukin (IL)-1α, IL-1β, IL-6, transforming growth factor (TGF)-β1, TGF-β2, and tumor necrosis factor-α are released by NSCs and show autocrine/paracrine effects^55^. Although it is not sure what kinds of factors are diminishing the proliferative effects and neurosphere forming effects of ONAS, it is obvious that NSCs response to the nanostructure when the density is low.

When we identified the mRNA expression of paracrine or autocrine factors including PDGFs, EGF, FGF2, TGF-β1, and TGF-β2 in NSCs cultured on either flat surface or ONAS (Supplementary Fig. S2), we did not find significant difference except *Pdgfc*. Since NSCs cultured on ONAS at low density are attached to each other but the NSCs on flat surface are distributed in a wide area as single cells, the concentration of the autocrine or paracrine factors released from neighboring cells would be higher and affect more in cells cultured on ONAS. If cells are plated at higher density, the total amount released from the cells increases and NSCs may not need to stick to each other for proliferation or migration. In addition, since *Pdgfc*, although the level is not much, was significantly up-regulated in NSCs cultured on ONAS, it may be responsible for the unique growth pattern observed in NSCs cultured on ONAS.

Generation of NSC colonies from a single cell has generally been performed on a Matrigel-coated surface^56,57^. In our study, low-density cells seeded on PDL and laminin coated ONAS generated compact colonies as neurospheres whereas the cells on the same material coated flat surface did not form any colonies. These data indicate that nanotopography alone can enhance the formation of  clonal colonies from single cells and suggest that ONAS can be used to generate a homogenous population of NSC clones from a single cell. Combined with recently developed single cell quantitative PCR and single cell RNA sequencing^58,59^, low density culture of NSCs on ONAS may provide accurate and precise transcriptome data of undifferentiated homogenous clonal NSCs. hESCs are also known to form colonies from single cells on Matrigel- or laminin-coated surfaces when mouse embryonic fibroblast conditioned media is used^56^. Similar to our results, plated on nanotopography, single hESCs showed enhanced colony formation without those previously reported media or coating conditions^20^.

The differential growth patterns seen between ONAS and the flat surface may also be due to reorganization of cytoskeletal proteins. It is reported that, on nanopillar surfaces with diameters ranging 120–170 nm, interaction between hESCs and nanotopography reinforces E-cadherin mediated cell-cell interactions and results in reduced formation of focal adhesion^54^. In the current study, NSCs cultured on the flat surface showed filopodia, finger-like membrane protrusion structures and lamellipodia. Lamellipodia are thin, sheet like projections formed at the leading edge of migrating cells, and are rich in actin filaments^60^. In contrast, NSCs cultured on ONAS did not show any membrane protrusion structures. Cell-cell interaction appeared to be tightened in NSCs cultured on ONAS at low density. Although different nanopatterns and cells were used in previous and current studies, in hESC cultures on nanopillar (diameter raging 120–170 nm) and NSCs in our study on nanopore ONAS (diameter: ~200 nm, depth: ~500 nm, and center-to-center distance: ~500 nm), the cells grew similarly. hESCs and NSCs formed compacted clones or spheres^54^. However, in our recent study, when ONAS was used to generate differentiated pancreatic cells, it induced filopodia formation on the edge of hESCs whereas flat control enhanced lamellipodia without filopodia^20^. These suggest that depending on culture condition and cell types, the response to nanostructure can be specific.

During cell migration, it is proposed that a cell first adheres to the substratum and then extends protrusions. It is reported that when adhesion is perturbed by using a blocking antibody, leukocytes show reduced spreading and protrusion^61^. Thus, it may be possible that, at clonal density, single NSCs do not adhere to the ONAS but increase interaction among the cells and proliferate as neurospheres. However, when the density is increased, NSCs adhere to the ONAS, possibly affected by the factors released by neighboring NSCs or cells. Our data provide evidence that proliferation, differentiation and migration of NSCs are affected by nanotopography and the current culture conditions of growing NSCs on flat surface need to be replaced to nanostructures to regulate NSC fate and behavior more precisely and economically. In addition, it appears that further studies on combined effects of growth factors and nanotopography on NSC behaviors using human NSCs are essential to generate specific types of cells from NSCs for the development of regenerative medicine.

Cells interplay with other cells or ECM via adhesion receptors such as integrins and cadherins^62^. Integrins play important roles in ECM mediated adhesion whereas cadherins mediate critical functions for adhesion between cells. Adhesion receptors like integrins and cadherins activate Rho, Rac and Cdc42 and remodel the actin cytoskeletons^63–65^. Cadherins bind to β-catenin, γ-catenin, p120 catenin family and so on^66^. Because cadherins bind to β-catenin, cadherins are considered to sequester β-catenin from Wnt signaling and block the downstream signaling^67^. It has also been reported that receptor tyrosine kinases are affected by cadherins. One study showed that E-cadherin mediated cell-cell adhesion inhibited EGF receptor (EGFR) activation but it is also known that E-cadherin is involved in the activation of EGFR and its downstream signals such as phosphatidylinositol 3-kinase and mitogen-activated protein kinase and results in tumor survival^68–70^. Interestingly, it has been known that down regulation of N-cadherin results in premature differentiation of NSCs^71^. It is also reported that a high level of N-cadherin is associated with symmetric division of NSCs^72^. We observed increased mRNA expression of E-cadherin in NSCs cultured on ONAS (Supplementary Fig. S3), and it may tighten the cell-cell contact and provide the unique proliferation feature of NSCs, such as growing as neurospheres. Although we could not detect any significant different N-cadherin mRNA expression levels between cells cultured on the flat surface and ONAS (data not shown), cells cultured on ONAS expressed significantly higher amount of E-cadherin and did proliferate better than those cultured on flat surface. Similar to our results, a recent study showed that overexpression of E-cadherin in NSCs increased their proliferation but reduced NSC migration^73^. We are not sure whether E-cadherin expression in NSCs cultured on ONAS is responsible for the increased proliferation on the first day, thus it would be interesting theme to investigate for future studies.

In summary, we have demonstrated increased proliferation and a distinct neurosphere forming growth pattern of NSCs on ONAS when cells were plated at low density. Our findings can be applied to culturing NSCs to obtain homogenous populations at clonal density. Integration of nanotopography on culture dishes may provide exciting opportunities to regulate NSC fate and behavior and to develop new therapeutic approaches for regenerative medicine.

## Methods

### Fabrication of PS cell-culture platforms with ONAS

For the generation of an ONAS on the AAO nano-template in a phosphoric acid bath, we used an anodization time of 3 h, an applied voltage of 195 V, a temperature of −5 °C. A nano-injection molding process (SE50D; Sumitomo, Tokyo, Japan) with the nano-mold insert was utilized for the production of the nano Petri dishes. PS (GP125EI; Kumho Petrochemical, Seoul, Korea), the commonly used thermoplastic material for cell-culture wares, was chosen as the substrate material for the nano Petri dish due to its validated research results in cell studies. Considering the replication quality of the nanopore arrays, the nano-injection molding was conducted at optimal conditions (a melting temperature of 220 °C, a mold temperature of 90 °C, an injection speed of 24 mm s^−1^, and a packing pressure of 120 MPa)^33^. In addition, conventional PS flat-surface Petri dishes, used as a control group, were also produced by injection molding with a mold insert having a mirror-like surface.

### Surface modification of nano Petri dishes

The surfaces of the nano and flat Petri dishes were treated by oxygen plasma (VITA I; Femto Science, Hwaseong, Korea), with a plasma power of 50 W, for 10 s before cell culture, as shown in Fig. 1F. The oxygen plasma treatment was found to enhance both hydrophilicity and cell attachment to the PS nano and flat Petri dishes^33^. The hydrophobic pristine PS flat and nanopore surfaces were found to have large values of CAs over 90° (SmartDrop; Femtofab, Pohang, Korea), whereas the plasma-treated surfaces were indicated to have small values of CAs of less than 60°; ergo, it appeared to be a good indicator for adequate cell attachment to the surface. Though the CA values increased with time, the CA values were stabilized around 60°, as shown in Fig. 2C and D.

### Surface sterilization and coating with poly-D-lysine and laminin

Flat and nano Petri dishes were sterilized by 70% ethanol immersion for 30 min and UV irradiation for 30 min. The substrates were coated with 0.01% PDL (Sigma-Aldrich, St. Louis, MO, USA) at room temperature for 4 h and washed with autoclaved water. The surfaces were then coated with 10 μg ml^−1^ laminin (Invitrogen, Carlsbad, CA, USA) at 37 °C for 5 h, and rinsed with Dulbecco’s modified Eagle’s medium/F12 (DMEM/F12; Gibco, Grand Island, NY, USA).

### Cell culture

Animal experiments were performed in accordance with Chung-Ang University and NIH standards of animal care and approved by Chung-Ang University animal care and use committee (Permit Number: 2014-00032, 2015-00002). NSCs were isolated from the cortex of E14 Sprague-Dawley rat (Orient Bio, Seongnam, Korea) embryos. After dissociation, NSCs were plated into tissue culture flasks (200,000 cells ml^−1^) and expanded as neurospheres at 37 °C/5% CO_2_ for 6 days in DMEM/F12 supplemented with 1% (v/v) PSA (Gibco), 2% (v/v) B27 (Gibco), 20 ng ml^−1^ EGF (Chemicon, Temecula, CA, USA), and 20 ng ml^−1^ FGF2 (Chemicon). The medium was replaced every 2 days. Neurospheres were then incubated with accutase (Chemicon) at 37 °C for 10 min to be dispersed into a single-cell suspension, and dissociated cells were plated onto flat and nano Petri dishes pre-coated sequentially with PDL and laminin.

### MTT assay

Cell proliferation was assessed using the 3-(4,5-dimethylthiazol-2-yl)-2,5-diphenyltetrazolium bromide (MTT; Sigma-Aldrich) assay. NSCs were seeded onto flat and nano Petri dishes at a density of 2 × 10^4^ cells cm^−2^ and cultured in the presence of EGF and FGF2 for 1 day. Then the MTT solution (final concentration, 1 mg ml^−1^) was injected to each plate and incubated at 37 °C for 1 h. The Formazan crystals formed in NSCs were solubilized with 20% sodium dodecyl sulfate (Amresco, Solon, OH, USA) in 50% aqueous *N*,*N*-dimethylformamide (Sigma-Aldrich). Lysates were transferred to 96-well plates (Corning, Corning, NY, USA), and absorbance was measured at 550 nm using the Synergy H1 Hybrid Multi-Mode Microplate Reader (BioTek, Winooski, VT, USA). The relative proliferation was expressed as percentage of [OD_nanopore_ − OD_blank_]/[OD_flat_ − OD_blank_] * 100.

### Immunocytochemistry and cell counting

Cells were fixed with 4% paraformaldehyde (PFA; USB Products, Cleveland, OH, USA) for 30 min and washed with phosphate-buffered saline (PBS). Fixed cells were blocked with 5% normal goat serum (Millipore, Temecula, CA, USA) and 0.2% Triton X-100 (Amresco) in PBS for 40 min. The cells were then incubated for more than 1.5 h with primary antibodies: anti-βIII tubulin antibody (TuJ1, mouse monoclonal antibody, 1:1000; Sigma-Aldrich), anti-GFAP (rabbit polyclonal antibody, 1:1000; Dako, Glostrup, Denmark), and anti-GalC (mouse monoclonal antibody, 1:500; Millipore). After rinsing with PBS, the cells were incubated for 1 h with secondary antibodies conjugated to Alexa Fluor 488 (goat anti-mouse immunoglobulin G [IgG], 1:1000; Invitrogen), or Cy3 (goat anti-rabbit IgG, 1:1000; Jackson ImmunoResearch, West Grove, PA, USA). 4′-6-diamidino-2-phenylindole (DAPI, 1:10000 in PBS; Sigma-Aldrich) was added for 5 min to stain the nuclei. The images were obtained using an inverse fluorescence microscope (DMIL; Leica, Wetzlar, Germany). To avoid a bias in measurement, the photos were randomly taken, and TuJ1-, GFAP-, GalC- or DAPI-positive cells were counted. The number of TuJ1-, GFAP- or GalC- positive cells was divided by that of DAPI-positive cells to obtain the percentage.

### Fluorescent staining of actin and focal adhesion

Cells were fixed with 4% PFA for 30 min, permeabilized for 10 min with 0.2% Triton X-100 in PBS, and blocked for 1 h with 2% bovine serum albumin (BSA; Millipore) and 0.2% Triton X-100 in PBS at room temperature. The cells were then incubated with anti-vinculin (mouse monoclonal antibody, 1:100; Sigma-Aldrich) overnight at 4 °C. After PBS rinses, the cells were incubated for 1 h with Alexa Fluor 546-conjugated secondary antibody (goat anti-mouse IgG1, 1:1000; Invitrogen) and Alexa Fluor 488-conjugated phalloidin (1:40; Invitrogen) at room temperature. The cells were washed 3 times with PBS and counterstained with DAPI for 5 min before acquiring fluorescence images using an inverse fluorescence microscope.

### Scanning electron microscope imaging

Cells were fixed with 2.5% glutaraldehyde (Sigma-Aldrich) in 0.1 M phosphate buffer for 2 h at room temperature, followed by rinsing with deionized water 3 times for 10 min each. The cells were dehydrated through ethanol series beginning with 30% and changing to solutions of 50%, 70%, 90%, and three times 100% for 10 min each. Dehydrated cells were air dried overnight, coated with platinum for 5 min and were then observed with FE-SEM (SIGMA HD; Carl Zeiss, Jena, Germany).

### Low-density experiments

NSCs were seeded onto flat and nano Petri dishes at a density of about 1111 cells cm^−2^ (3.3 cells μl^−1^). The cells were cultured in the presence of EGF and FGF2, and the medium was replaced at 3 days post-seeding. Five days after proliferation, NSCs were differentiated in the absence of EGF and FGF2 for 3 days. After fixation with 4% PFA for 30 min, immunocytochemistry was performed as described above. Digital images of cell cultures were obtained every day at the same spots using JuLI-Digital bio (NanoEnTek, Seoul, Korea).

### Statistical analysis

Values were expressed as means ± standard error of mean (s.e.m.), and statistical significance was determined using Student’s *t*-test (*P < 0.05 vs. control).

## Electronic supplementary material

Supplementary Information

Supplementary Video S1

Supplementary Video S2
