# Supplementary material for: Cell density-dependent differential proliferation of neural stem cells on omnidirectional nanopore-arrayed surface
Source: Sci Rep. 2017 Oct 12;7:13077. doi: 10.1038/s41598-017-13372-6 (PMC5638797; doi:10.1038/s41598-017-13372-6)
Supplement: Supplementary file 1 — Supplementary Information [file 41598_2017_13372_MOESM1_ESM.pdf]

Supplementary Information

Cell density-dependent differential proliferation of neural stem cells on omnidirectional nanopore-arrayed surface

Kyoung Je Cha [1,2,#], Sun-Young Kong [3,#], Ji Soo Lee [3], Hyung Woo Kim [1], Jae-Yeon Shin [3], Moonwoo La [1,4], Byung Woo Han [5], Dong Sung Kim [1,*] & Hyun-Jung Kim [3,*]

[1]Department of Mechanical Engineering, Pohang University of Science and Technology (POSTECH), San 31 Hyoja-dong Nam-gu, Pohang 790-784, South Korea.

[2]Present address: Ultimate Manufacturing Technology Group, Korea Institute of Industrial Technology (KITECH), Techno sunhwan-ro Yuga-myeon Dalseong-gun, Deagu 711-880, South Korea.

[3]Laboratory of Molecular and Stem Cell Pharmacology, College of Pharmacy, Chung-Ang University, 221 Heukseok-dong Dongjak-gu, Seoul 156-756, South Korea.

[4]Present address: Molds & Dies R&D Group, Korea Institute of Industrial Technology (KITECH), 156 Gaetbeol-ro, Yeonsu-gu, Incheon 406-840, South Korea

[5]Department of Biochemistry, College of pharmacy, Seoul National University, San 56-1 Sillim-dong Gwanak-gu, Seoul 151-742, South Korea.

[#]These authors contributed equally to this work.

[*]Correspondence and requests for materials should be addressed to D.S.K. (email: smkds@postech.ac.kr) or H.-J.K. (email: hyunjungkim@cau.ac.kr)

Supplementary Video S1 and S2

Time-lapse video of NSCs grown in the presence of mitogens on the flat surface **(Video S1)** or ONAS **(Video S2)** at clonal density (3.3 cells μl^{-1}). NSCs were expanded for 1 week as neurospheres, dissociated and seeded on the flat surface for 24 h, and then the images were acquired every 10 min for additional 48 h.

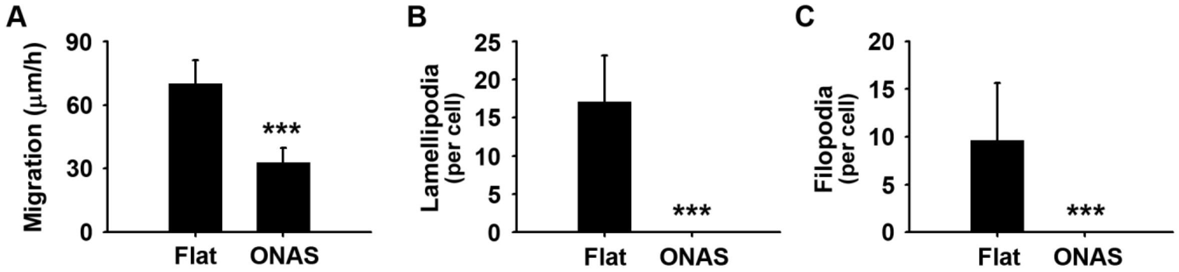

Supplementary Figure S1. Migration and formation of lamellipodia/filopodia of NSCs cultured at clonal density (3.3 cells μl^{-1}).

(A) Quantification of cell migration. After 1 week of expansion as neurospheres, NSCs were dissociated and cultured on the flat surface or ONAS in the presence of mitogens at 3.3 cells μl^{-1}. Then, migration distance of individual cells was measured for 4 h (68 h–72 h after plating). The rate of migration was calculated as the total migration distance divided by the total time taken (4 h). Results were presented as mean ± s.d. (n = 10). ***$P < 0.001$, Student's *t*-test. **(B and C)** Quantification of lamellipodia **(B)** and filopodia **(C)** in phalloidin-stained cells (as shown in Fig. 6C). NSCs grown in the presence of mitogens for 4 days were fixed, stained with phalloidin, and the number of lamellipodia and filopodia formed per cell was counted. Values were mean ± s.d. (n = 17). ***$P < 0.001$, Student's *t*-test.

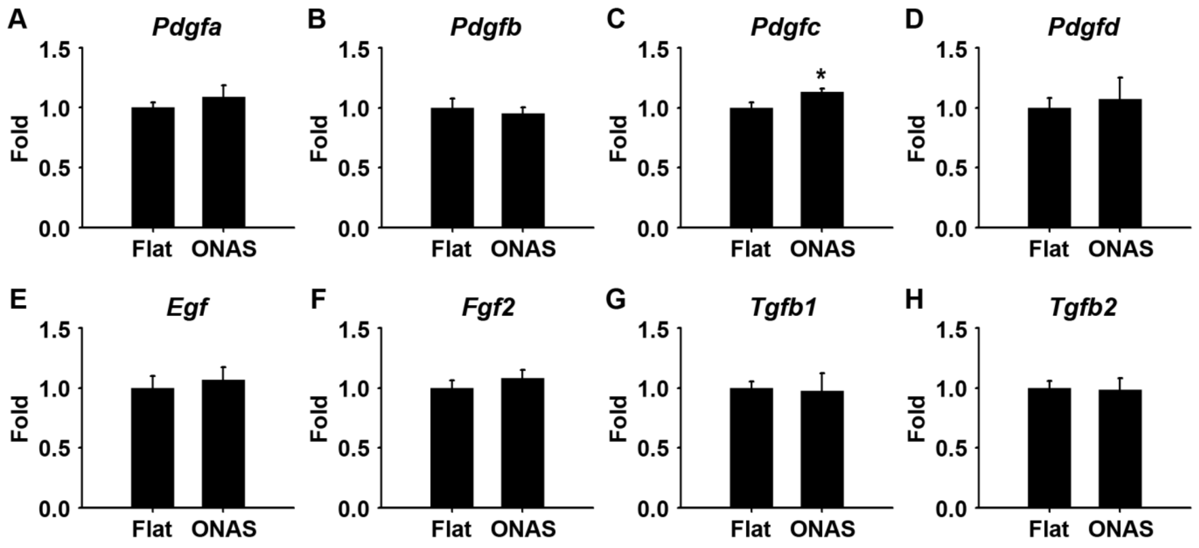

Supplementary Figure S2. The gene expression of autocrine/paracrine factors in proliferating NSCs at clonal density (3.3 cells μl^{-1}).

NSCs were expanded for 1 week as neurosphere, dissociated onto the flat surface or ONAS at 3.3 cells μl^{-1}. The mRNA levels of *Pdgfa* **(A)**, *Pdgfb* **(B)**, *Pdgfc* **(C)**, *Pdgfd* **(D)**, *Egf* **(E)**, *Fgf2* **(F)**, *Tgfb1* **(G)**, and *Tgfb2* **(H)** were evaluated by real-time RT PCR in NSCs grown for 4 days in the presence of mitogens. The expression level of each mRNA was normalized to that of *Gapdh*. Data were expressed as mean ± s.e.m. (n = 3). *P < 0.05, Student's *t*-test.

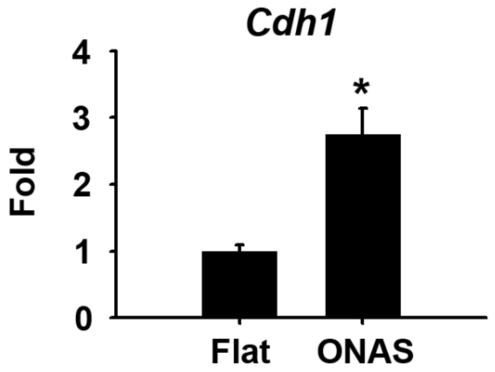

Supplementary Figure S3. The *Cdh1* expression in proliferating NSCs at clonal density (3.3 cells μl^{-1}).

After 1 week of expansion as neurospheres, NSCs were dissociated and plated onto either the flat surface or ONAS at 3.3 cells μl^{-1}, and grown in the presence of mitogens for 4 days. The mRNA expression levels of *Cdh1* were determined by real-time RT PCR. *Gapdh* was used as the internal control. Data were expressed as mean ± s.e.m. (n = 3). *P < 0.05, Student's *t*-test.

Supplementary Methods

Real-time RT PCR. Total RNA was extracted using the TRIzol reagent (Invitrogen, Carlsbad, CA, USA). First-strand cDNA was synthesized from 1 μg of total RNA using a QuantiTect Reverse Transcription Kit (Qiagen, Limburg, Netherlands). Real-time PCR was performed using iQ SYBR Green supermix (Bio-Rad, Hercules, CA, USA), using cycling conditions as follows: initial activation at 95°C for 3 min, followed by 40 cycles of denaturation at 95°C for 10 s, annealing at 58°C for 15 s, and extension at 72°C for 20 s. The primer sets used to amplify cDNA are described below. The housekeeping gene *Gapdh* was used as an internal control.

**Target**	**Forward primer (5'–3')**	**Reverse primer (5'–3')**
Cdh1	ATGGTTCACCCATTGCCACT	CGGGTTTCTCTGGCAGAACT
Pdgfa	TGTCAAGGTGGCCAAAGTGG	CTCCTCCTCCCGATGGTCTG
Pdgfb	CCGAGCACATTCTGGAGTCG	GGTGCGATCGATGAGGTTCC
Pdgfc	GTGCGGATCCAGCTGACATT	ACAGTCCCAGAACCACACCA
Pdgfd	GCGTTACAGTTGCACTCCCA	AACCACAGTTGCCACCACAG
Egf	CCTGACATCAGATGGTCCTC	ATCACATTCCCAGGATGCTA
Fgf2	TGGCTATGAAGGAAGATGGA	TCAGTGCCACATACCAACTG
Tgfb1	CGGACTACTACGCCAAAGAA	TTCCCGAATGTCTGACGTAT
Tgfb2	AATGTTGTTGCCCTCCTACA	GCAATTATCCTGCACATTCC
Gapdh	AGTTCAACGGCACAGTCAAG	GTGGTGAAGACGCCAGTAGA

Statistical analysis. Values were expressed as mean ± standard error of mean (s.e.m.) or standard deviation (s.d.). Statistical significance was determined using Student's *t*-test (*P < 0.05 and ***P < 0.001 vs. control).
